# Topographic expression of the Hippo transducers TAZ and YAP in triple-negative breast cancer treated with neoadjuvant chemotherapy

**DOI:** 10.1186/s13046-016-0338-7

**Published:** 2016-04-02

**Authors:** Patrizia Vici, Cristiana Ercolani, Anna Di Benedetto, Laura Pizzuti, Luigi Di Lauro, Francesca Sperati, Irene Terrenato, Teresa Gamucci, Clara Natoli, Franco Di Filippo, Claudio Botti, Maddalena Barba, Marcella Mottolese, Ruggero De Maria, Marcello Maugeri-Saccà

**Affiliations:** Division of Medical Oncology B, “Regina Elena” National Cancer Institute, Rome, Italy; Department of Pathology, “Regina Elena” National Cancer Institute, Rome, Italy; Biostatistics-Scientific Direction, “Regina Elena” National Cancer Institute, Rome, Italy; Medical Oncology Unit, ASL Frosinone, Frosinone, Italy; Department of Experimental and Clinical Sciences, University “G. d’Annunzio”, Chieti, Italy; Department of Surgery, “Regina Elena” National Cancer Institute, Rome, Italy; Scientific Direction, “Regina Elena” National Cancer Institute, Rome, Italy; Division of Medical Oncology B and Scientific Direction, “Regina Elena” National Cancer Institute, Via Elio Chianesi 53, 00144 Rome, Italy

**Keywords:** Hippo pathway, TAZ, YAP, Triple-negative breast cancer, Stromal cells

## Abstract

**Background:**

The Hippo signaling acts as a tumor-suppressor pathway that negatively regulates TAZ and YAP. Increasing evidence supports the activation of TAZ and YAP in breast cancer. Moreover, the Hippo pathway is involved in the biology of non-neoplastic cells residing in the tumor microenvironment. On this basis, we herein assessed TAZ and YAP in triple-negative breast cancer and its surrounding microenvironemnt in order to investigate their impact on pathological complete response (pCR) and tumor recurrence.

**Methods:**

Sixty-one triple-negative breast cancer patients treated with neoadjuvant chemotherapy were retrospectively evaluated. TAZ and YAP were assessed by immunohistochemistry and classified as positive or negative according to the percentage of tumor-expressing cells, cellular localization, and staining intensity. TAZ and YAP expression was also evaluated in non-lymphocytic stromal cells, tumor-infiltrating lymphocytes (TILs) and endothelial cells. The Pearson’s Chi-squared test of independence was used to test the association between TAZ/YAP and clinical-molecular factors. A multivariate logistic regression model was generated to identify variables impacting pCR. The Kaplan-Meier method and the log-rank test were used for estimating and comparing survival curves. Cox proportional regression models were built to evaluate the risk of recurrence for the variables considered. Internal validation was carried out with a re-sampling without replacement method.

**Results:**

We did not observe any impact on pCR rate when TAZ and YAP were addressed singularly. Conversely, the combined expression of YAP in tumor cells and non-lymphocytic stromal cells was an independent predictor of reduced pCR rate in the multivariate model (OR 7.13, 95 % CI: 1.23–41.41, *p* = 0.029). Next, the combined expression of TAZ and YAP was associated with shorter disease-free survival (DFS) in multivariate analysis (HR 3.07, 95 % CI: 1.24–7.61, *p* = 0.016). The robustness of these findings were internally validated.

**Conclusions:**

The combined expression of YAP in TNBC cells and in the surrounding stroma seems to be associated with a decreased likelihood to achieve pCR. Conversely, the combined expression of TAZ and YAP in tumor cells conferred poor survival outcomes.

**Electronic supplementary material:**

The online version of this article (doi:10.1186/s13046-016-0338-7) contains supplementary material, which is available to authorized users.

## Background

The Hippo pathway is a regulator of tissue growth that in neoplastic diseases is considered a tumor suppressor signaling [1]. The Hippo core module, composed by the kinases MST1, MST2, LATS1, LATS2 and the adaptor proteins SAV1, MOB1A, MOB1B, carries out an inhibitory phosphorylation of the Hippo transducers TAZ and YAP. When the pathway is switched off, or when pathway-extrinsic cues activate TAZ and YAP, they translocate to the nucleus [2]. Here, after interactions with other factors, TAZ and YAP promote the transcription of target genes [2].

In breast cancer (BC), the activation of the TAZ/YAP-transcriptional program feeds a number of tumor-promoting functions [3]. An important oncogenic function of TAZ relates to its association with BC stem cells (BCSCs) [4, 5]. Cordenonsi et al. first reported on the connection between TAZ and self-renewal of BCSCs [4]. Our group enforced this link [5]. Characterization of a collection of patient-derived BCSCs and related xenografts enabled us to mechanistically describe the involvement of TAZ in chemoresistance and metastatic spread [5]. Conversely, the involvement of YAP in BC is ambiguous, considering that both tumor-promoting and tumor-suppressive functions have been proposed [3]. These latter are based on preclinical evidence describing a negative regulation of YAP mediated by AKT [6], and the interaction between YAP and p73 that leads to the transcription of proapoptotic genes [7].

Beyond cancer cells, the Hippo pathway is involved in the biology of other cell types residing in the tumor microenvironment. Maintenance of cancer-associated fibroblast (CAFs) properties was tied to YAP activation [8]. Moreover, a non-canonical, immune-related Hippo/MST pathway orchestrates activation, trafficking, and homing of T cells [9].

The growing body of evidence connecting TAZ and YAP to BC biology prompted us to translate preclinical findings into clinical investigations. As a general principle, the focus was placed on the neoadjuvant setting, which is increasingly exploited for the identification of cancer biomarkers. This is rooted in the link between an intermediate endpoint, namely pathological complete response (pCR) after neoadjuvant chemotherapy (NACT), and survival outcomes [10]. In the HER2-positive background, we have already reported on a significant association between elevated TAZ expression and pCR in Luminal-type/HER2-positive tumors [11].

Herein we present results from triple-negative BC (TNBC), the most aggressive BC form. TAZ and YAP were assessed by immunohistochemistry in pretreatment biopsies related to 61 stage II-III TNBC patients who received anthracycline-taxane-based NACT. TAZ and YAP were assessed in tumor cells, and in non-neoplastic cells including endothelial cells, non-lymphocytic stromal cells, and tumor-infiltrating lymphocytes (TILs).

This study was planned with the following goals: i) describing the topographic expression of TAZ/YAP in TNBC, ii) investigating the association between TAZ/YAP and pCR, iii) exploring the connection between TAZ/YAP and survival outcomes, and iv) providing clues on the role of YAP as an oncogene or an oncosuppressor in TNBC.

## Methods

### Study participants and procedures

Sixty-one TNBC patients treated with NACT were included in this retrospective analysis. Patients were considered eligible if NACT was completed, data were available on clinical-pathological features including stage, estrogen receptor (ER) status, progesterone receptor (PgR) status, tumor grade, Ki-67 and pCR, and tumors did not show HER2 overexpression/amplification according to ASCO-CAP guidelines. In this analysis, six patients with weak expression (≤10) of either ER or PgR in diagnostic biopsies were included, given that in these tumors hormone receptor status switched from weak positivity to negativity in residual cancers. For these tumors, a basal-like molecular portrait may be hypothesized in light of the fact that up to 20 % of basal-like cancers express the ER [12]. Stromal TILs were assessed as recently reported by the International TILs Working Group [13].

NACT consisted in anthracycline-taxane-based chemotherapy regimens, either with a concomitant or sequential schedule, as detailed elsewhere [14]. Seven patients received adjuvant chemotherapy. pCR was defined as no residual invasive tumor in both breast and axilla, irrespective of the presence of ductal carcinoma in situ (ypT0/is ypN0).

While the impact of TAZ/YAP on pCR rate was evaluated in the entire cohort (*N* = 61), the impact of TAZ/YAP expression on survival outcomes was evaluated in 57 patients, considering that we were unable to retrieve this information for four patients. Disease-free survival (DFS) was defined as time from diagnosis until locoregional, invasive contralateral or distant recurrence, or death due to any cause.

The immunohistochemical assessment of TAZ and YAP was performed in formalin-fixed paraffin-embedded tissues using the monoclonal antibody (MoAb) anti-TAZ (M2-616, BD Pharmingen) at the dilution of 1:400 and the MoAb anti-YAP (H-9, Santa Cruz) at the dilution of 1:200.

For the evaluation of TAZ and YAP in cancer cells, their expression was reported both in terms of percentage of tumor-expressing cells and staining intensity (0 = absent, 1+ = weak, 2+ = moderate, and 3+ = strong). For tumors with both nuclear and cytoplasmic expression, staining intensity and percentage of tumor-expressing cells were independently assessed in, and reported for, the two cellular compartments.

Tumors were classified as negative (TAZ^neg^, YAP^neg^) or positive (TAZ^pos^, YAP^pos^) on the basis of their cellular localization, staining intensity, and percentage of tumor-expressing cells. TAZ/YAP positivity was defined as a distinct, moderate (2+) or strong (3+) nuclear immunoreactivity in ≥10 % of neoplastic cells [5].

For the evaluation of TAZ/YAP in the tumor microenvironment, the three main cellular components, namely endothelial cells, non-lymphocytic stromal cells and TILs, were morphologically identified. For each compartment, TAZ/YAP expression was considered positive when cells exhibited a distinct homogeneous/heterogeneous immunoreactivity, irrespectively of the subcellular localization. Faintly staining cells or positive cells located in the tumor margin or in areas with poor morphology were not included in the analysis. The related molecular variables were designated as following: TAZ^stroma^ and YAP^stroma^, TAZ^TILs^ and YAP^TILs^, TAZ^end^ and YAP^end^.

Two investigators (ADB and CE) blinded to treatment outcomes independently evaluated immunoreactivity. Discordant cases were further reviewed by a third observer (MM).

This retrospective study has been conducted in accordance with the Declaration of Helsinki and approved by the Ethic Committee of “Regina Elena” National Cancer Institute of Rome, the coordinating centre. Written informed consents were secured before chemotherapy from any single participant.

### Statistical analysis

Descriptive statistics were used to summarize study participants’ characteristics. Continuous data were reported as mean and standard deviation, and categorical data by frequencies and percentage values.

The Pearson’s Chi-squared test of independence (2-tailed) and the Fisher Exact test, when appropriate, were used to assess the relationship between TAZ/YAP and clinical-molecular features and pCR. Survival curves were estimated with the Kaplan-Meier method, and the log-rank test was used for comparisons.

To identify independent predictors of pCR, a multivariate logistic regression model was generated with variables that tested significant at the univariate assessment, and the related estimates reported as Odds Ratio (OR) and 95 % Confident Interval (CI). To identify independent predictors of DFS, multivariate Cox proportional hazard models were built with the same modality, and the related estimates reported in terms of Hazard Ratios (HR) and 95 % CI.

The risk to obtain an overfitting multivariate model for DFS was controlled through a re-sampling without replacement technique, envisioning the generation of 100 less-powered datasets obtained by randomly removing ~20 % of the original sample. For each simulation, the Cox model was repeated and the replication rate was calculated.

We considered statistically significant p values less than 0.05. Statistical analyses were carried out using SPSS software (SPSS version 21, SPSS Inc., Chicago, IL, USA).

## Results

Clinical-pathological features and treatment outcomes related to the 61 TNBC patients included in this study are summarized in Table 1. All the bioptic samples examined in this study contained at least 30 % of neoplastic cells.

**Table 1:** Baseline characteristics and treatment outcome of TNBC patients treated with neoadjuvant chemotherapy (*N* = 61)

Characteristics	*N* (%)
Age at diagnosis	
Mean ± SD	49.8 ± 11.4
Median (min-max)[IQrange]	48.4 (25.6–76.6) [44.3–58.3]
Stage	
II	21 (34.4)
III	40 (65.6)
Ki-67	
Mean ± SD	58.2 ± 24.7
Median (min-max)[IQrange]	60 (10–90) [40–80]
Grade	
1–2	22 (36.1)
3	39 (63.9)
Chemotherapy	
Sequential	52 (85.2)
Concomitant	9 (14.8)
Pathological complete response	
Yes	18 (29.5)
No	43 (70.5)

As aforementioned, activation of TAZ/YAP promotes multiple oncogenic function in BC cells [3]. TAZ/YAP have also been linked to the function of non-neoplastic cells residing in the tumor microenvironment, and potentially affecting therapeutic resistance and survival outcomes. An immune-related, non-canonical Hippo/MST pathway is emerging as a multifaceted regulator of adaptive immunity, being essential for proper T cell development and function [9]. Next, TAZ/YAP are central for survival of endothelial cells in response to changes in cell geometry [2]. Finally, YAP was reported as a key factor for the maintenance of CAFs that, in turn, have been associated with a variety of tumor-promoting functions, even including resistance to chemotherapy [8]. On this basis, we investigated the impact of TAZ/YAP on therapeutic outcomes by considering their topographic expression, namely their presence/absence in cancer cells, non-lymphocytic stromal cells, endothelial cells, and TILs.

**Fig. 1:**
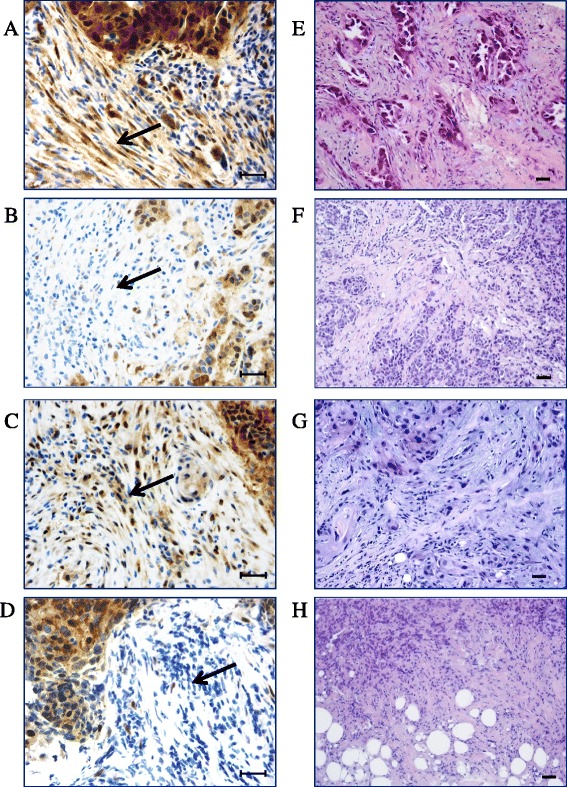
Representative examples of immunohistochemical expression of TAZ and YAP in TNBC patients. **a** a tumor expressing TAZ in tumor cells and in non-lymphocytic stromal cells. **b** a tumor expressing TAZ exclusively in cancer cells. **c** a tumor expressing YAP in tumor cells and in non-lymphocytic stromal cells. **d** a tumor expressing YAP in tumor cells, but not in non-lymphocytic stromal cells. *Black arrows* indicate the stromal compartment. The corresponding H&E staining are also showed (**e**–**h**). Scale bar = 30 μm

The expression of TAZ and YAP in tumor cells (and their cellular localization), non-lymphocytic stromal cells, endothelial cells, and TILs is summarized in Additional file 1: Table S1. Representative immunohistochemical staining patterns are illustrated in Fig. 1.

When TAZ and YAP were evaluated for their association with established clinical-molecular features and pCR, the only associations that resulted significant were between TAZ^pos^ and higher stage (*p* = 0.030) and YAP^pos^ and higher grade (*p* = 0.028) (Additional file 2: Table S2). Thus, neither TAZ nor YAP, independently on whether they were considered in tumor cells or in non-neoplastic cells, showed a significant association with pCR when singularly considered.

We therefore investigated whether the expression of TAZ or YAP in cancer cells, together with their expression in non-cancerous cells, was able to identify the category of patients with reduced pCR rate. As reported in Table 2, the YAP^pos^/YAP^stroma^ phenotype was significantly associated with reduced pCR rate at the uni- and multivariate assessment (OR 5.23, 95 % CI: 1.06–25.70, *p* = 0.042, and OR 7.13, 95 % CI: 1.23–41.41, *p* = 0.029, respectively).

**Table 2:** Univariate and multivariate logistic regression models evaluating the impact of the YAP^pos^/YAP^stroma^ phenotype on pCR (*N* = 61)

		Univariate regression model		Multivariate regression model^a^	
		OR (95 % CI)	*p*-value	OR (95 % CI)	*p*-value
Age	>49 vs ≤49	5.35 (1.51–19.03)	0.010	7.57 (1.81–31.65)	0.006
Stage	III vs II	0.65 (0.19–2.16)	0.481		
Grade	III vs II	1.66 (0.54–5.12)	0.380		
Ki-67	≥60 vs <60	0.23 (0.07–0.76)	0.016	0.24 (0.06–0.94)	0.041
YAP^pos/^YAP^stroma^	YAP^pos^/YAP^stroma^ vs other combinations	5.23 (1.06–25.70)	0.042	7.13 (1.23–41.41)	0.029

^a^Adjusted for Age, Ki-67 and YAP^pos^/YAP^stroma^

**Fig. 2:**
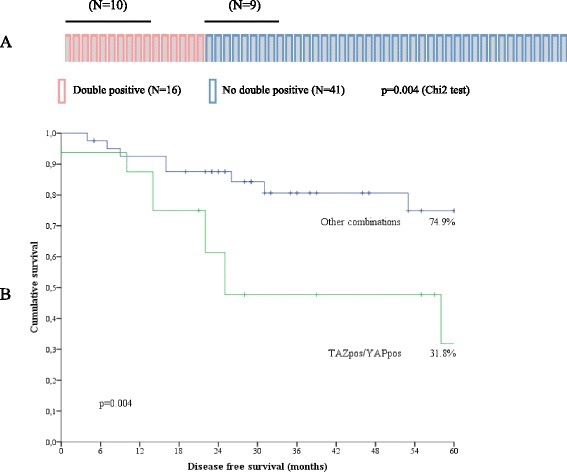
Impact of the combined expression of TAZ and YAP on tumor recurrence. **a** OncoPrint showing the individual distribution of tumor recurrences according to the combined nuclear expression of TAZ and YAP. Relapsed cases are indicated with the black line placed above the OncoPrint. **b** Kaplan-Meier survival curves regarding disease-free survival in TAZ^pos^/YAP^pos^ tumors compared with the negative counterparts

We next investigated whether TAZ and/or YAP impacted DFS. At a median follow-up of 31 months, 19 events were recorded in the 57 evaluable patients. As previously specified in the “Study Participants and procedures” section, post-surgical follow up data were not available for four patients. The pattern of disease recurrence is shown in Additional file 3: Table S3. Visceral and/or skeletal metastasis developed in 11 out of the 19 relapsed patients, and two patients had brain metastases. Again, neither TAZ nor YAP were associated with DFS when individually analyzed (data available upon request). Reasoning that, analogously to pCR rate, the combination of different markers may have been more informative, we then tested different biomarker combinations. With this strategy, we observed that the co-expression of TAZ and YAP in tumor cells (TAZ^pos^/YAP^pos^) was associated with tumor recurrence (*p* = 0.004; panel a in Fig. 2), and that these patients exhibited shorter DFS (*p* = 0.004; panel b in Fig. 2). Consistently, as shown in Table 3, the TAZ^pos^/YAP^pos^ phenotype was associated with an increased risk of relapse at the univariate analyses (HR 3.44, 95 % CI: 1.39–8.48, *p* = 0.007), and it was the only significant variable at the multivariate assessment (HR 3.07, 95 % CI: 1.24–7.61, *p* = 0.016). The consistency of the TAZ^pos^/YAP^pos^ model was internally validated using a re-sampling without replacement method. The replication rates, defined as the percentage of Cox regression models that yielded statistically significant results upon 100 replications in less-powered datasets (−20 % compared with the original one), were 96 and 66 % with statistical significance set at *p* <0.05 and *p* <0.01, respectively.

**Table 3:** Uni- and multivariate Cox regression models of disease-free survival in TNBC patients (*N* = 57) considering a model of double positivity for TAZ and YAP

		Univariate regression model		Multivariate regression model^a^	
		HR (95 % CI)	*p*-value	HR (95 % CI)	*p*-value
Age	>49 vs ≤49	1.17 (0.47–2.88)	0.738		
Stage	III vs II	1.17 (0.44–3.09)	0.751		
Grade	III vs I-II	1.71 (0.65–4.52)	0.278		
pCR	no vs yes	4.36 (1.00–18.99)	0.050	3.81 (0.87–16.71)	0.076
Ki-67	≥60 vs <60	0.44 (0.16–1.17)	0.099		
TAZ/YAP	TAZ^pos^/YAP^pos^ vs other combinations	3.44 (1.39–8.48)	0.007	3.07 (1.24–7.61)	0.016

^a^Adjusted for pCR and TAZ/YAP

## Discussion

In the present analysis we reported on the predictive and prognostic significance of TAZ and YAP expression, assessed both at the tumor and the microenvironment level, in a moderately-sized cohort of TNBC treated with NACT. Results from this pilot study suggested that TAZ/YAP expression might impact on both pCR rate and long-term survival outcomes, and underscore the complexity of the Hippo biology, which needs to be carefully considered in the search for Hippo-related prognostic and predictive biomarkers.

To our knowledge, this is the first report evaluating TAZ and YAP jointly in TNBC and its microenvironment and, more importantly, with a clear focus on pCR and recurrence [15–17]. We are aware that our findings are hypothesis-generating considering the retrospective design of this study. Nevertheless, our results raised some important considerations.

First, in BC great attention was put toward the identification of biomarkers related to the interactions of cancer cells with neighbor non-neoplastic cells. A number of microenvironment-related biomarkers have been proposed over the past decade, spanning from a wound-response signature denoting the transcriptional response of normal fibroblasts to serum [18] to the assessment of stromal TILs in the neoadjuvant setting [19]. Reasoning that the Hippo pathway is extensively associated with the function of non-neoplastic cells involved in the so-called tumor-stroma interplay, we envisioned a role for the Hippo signal in this process. Consistently, the identification of an association between YAP expression in both cancer cells and non-lymphocytic stromal cells and reduced pCR rate provides clinical ground for the feed-forward, self-reinforcing loop necessary for maintaining the CAF phenotype via YAP activation [8], and further highlights the role of CAFs in chemotherapy resistance [20].

Second, given the exploratory nature of this study we exclusively focused on Hippo transducers. Nevertheless, a number of stimuli intersect the Hippo cascade, which either encourage or restrain TAZ/YAP nuclear localization and gene transcription. To appropriately address this issue, a study where TAZ and YAP are evaluated together with further candidate biomarkers has been planned, envisioning the concomitant assessment of i) TAZ/YAP targets (Axl and CTGF [2]), ii) Hippo-dependent and independent cues that feed TAZ/YAP activation, including mechanisms involved in cell-cell adhesion and apical-basal polarity [2], mechanotransduction [21], RHO GTPases and the mevalonate pathway [22], and Wnt signaling [23], and iii) Markers of specific cellular components in the tumor microenvironment (e.g. CAFs, immune cell subsets). Due to the limited amount of biological materials available after extensive routine pathological assessment, we were unable to consider further biomarkers for this study. Thus, a wider pathway analysis that might enable us to identify a unique Hippo-related biomarker profile able to inform on both pCR and survival outcomes was recently initiated.

Another aspect that deserves mention relates to the biological significance of TAZ and YAP in BC. In this setting, TAZ has been defined as an oncogene promoting neoplastic transformation [24], chemotherapy resistance [5, 25], EMT [4, 26], cancer stem cell (CSC) self-renewal [4, 5], luminal to basal lineage switch [27], and distant dissemination [5, 28]. Conversely, in preclinical BC models YAP activation has been tied to opposite functions. On the one hand, YAP was found to promote neoplastic transformation [29–31], EMT [29], anchorage-independent growth, retention of the cancer-associated fibroblast (CAF) phenotype [8], and metastatization [32, 33]. On the other hand, tumor-suppressive activities have been reported, such as the interaction with p73 [7], its negative regulation mediated by oncogenic AKT and miR-200 [6, 34], and frequent loss of heterozygosity at 11q22.2 [35]. Our study was not designed to provide mechanistic insights into the molecular cues that modulate YAP activation in TNBC. Nevertheless, our results provide evidence in support of the concept that, at least in TNBC, YAP may elicit oncogenic functions. It is possible that YAP acts in different way in distinct BC molecular subtypes [3]. Indeed, Lehn et al. have recently reported on the association between low YAP expression and decreased recurrence-free survival in luminal A tumors [17]. Our results suggest an opposite interaction with pCR and long-term outcomes in TNBC, and underlie the risk of carrying out analyses in molecularly-unselected BC cohorts.

Intriguingly, survival data (DFS) raised the hypothesis that, in TNBC, both TAZ and YAP are involved, and possibly cooperate, in metastatic dissemination. A plausible explanation is that, albeit TAZ and YAP are closely related proteins, they elicit a partial different array of oncogenic activities. Consistently, the nature of up-regulated genes upon their forced expression in cell lines is partly different [36]. This is not surprising when considering the number of their transcriptional partners that, beyond TEAD transcription factors, also include SMAD and RUNX proteins [2]. Indeed, a partial different interaction of TAZ/YAP with the aforementioned transcriptional partners have been hypothesized [37], and biochemical studies reported differences in the way TAZ and YAP interact with TEAD4 [38].

Next, we have recently discussed possible strategies for assessing TAZ and YAP by IHC [3]. In our opinion, cytosolic expression should not be ignored, especially when evaluation at different time points is not feasible. Indeed, nuclear translocation of TAZ and YAP is dictated by multiple cues and arguable oscillates over time [2]. Nevertheless, in the present report we considered tumors as positive or negative mostly on the basis of cellular localization. This is due to the fact that the majority of the samples (~90 %) showed immunoreactivity in either the nucleus or the cytoplasm (Additional file 1: Table S1). Moreover, our study underlies the concept that the assessment of TAZ and YAP should be extended to the non-neoplastic compartment, in order to take into account the biological relevance of the Hippo pathway in CAFs, TILs, and endothelial cells.

Finally, the growing interest of our group toward the Hippo pathway in BC has fuelled a wave of studies in other BC subtypes, such as hormone receptor-positive/HER2-negative and hormone receptor-positive/HER2-positive BC, and in male BC. Results from these studies will provide a more exact picture on the clinical significance of Hippo transducers in individual BC subtypes.

## Conclusions

The combined expression of the Hippo transducer YAP in tumor cells and non-lymphocytic stromal cells seemed associated with reduced efficacy of anthracycline-taxane-based NACT in TNBC patients in terms of pCR rate. Moreover, the nuclear co-expression of TAZ and YAP may confer an increased risk of recurrence. Thus, Hippo-related biomarkers deserve larger studies in TNBC.

## Additional files

Additional file 1: Table S1. Expression of TAZ and YAP in cancer cells, non-lymphocytic stromal cells, endothelial cells, and tumor-infiltrating lymphocytes. Subcellular localization of TAZ/YAP in tumors is also reported (*N* = 61). (DOCX 13 kb) Additional file 2: Table S2. associations between TAZ/YAP, assessed in the tumor and in the microenvironment, and clinical-molecular features and pCR (*N* = 61). (DOCX 12 kb) Additional file 3: Table S3. Pattern of recurrence in the 19 TNBC patients. (DOCX 12 kb)

## Abbreviations

BC: breast cancer
BCSCs: breast cancer stem cells
CAFs: cancer-associated fibroblasts
CSCs: cancer stem cells
DFS: disease-free survival
EMT: epithelial-to-mesenchymal transition
ER: estrogen receptor
NACT: neoadjuvant chemotherapy
pCR: pathological complete response
PgR: progesterone receptor
TILs: tumor-infiltrating lymphocytes
TNBC: triple-negative breast cancer

## References

[1] Johnson R, Halder G (2014). The two faces of Hippo: targeting the Hippo pathway for regenerative medicine and cancer treatment. Nat Rev Drug Discov.

[2] Piccolo S, Dupont S, Cordenonsi M (2014). The biology of YAP/TAZ: hippo signaling and beyond. Physiol Rev.

[3] Maugeri-Saccà M, Barba M, Pizzuti L, Vici P, Di Lauro L, Dattilo R (2015). The Hippo transducers TAZ and YAP in breast cancer: oncogenic activities and clinical implications. Expert Rev Mol Med.

[4] Cordenonsi M, Zanconato F, Azzolin L, Forcato M, Rosato A, Frasson C (2011). The Hippo transducer TAZ confers cancer stem cell-related traits on breast cancer cells. Cell.

[5] Bartucci M, Dattilo R, Moriconi C, Pagliuca A, Mottolese M, Federici G (2015). TAZ is required for metastatic activity and chemoresistance of breast cancer stem cells. Oncogene.

[6] Basu S, Totty NF, Irwin MS, Sudol M, Downward J (2003). Akt phosphorylates the Yes-associated protein, YAP, to induce interaction with 14-3-3 and attenuation of p73-mediated apoptosis. Mol Cell.

[7] Strano S, Monti O, Pediconi N, Baccarini A, Fontemaggi G, Lapi E (2005). The transcriptional coactivator Yes-associated protein drives p73 gene-target specificity in response to DNA Damage. Mol Cell.

[8] Calvo F, Ege N, Grande-Garcia A, Hooper S, Jenkins RP, Chaudhry SI (2013). Mechanotransduction and YAP-dependent matrix remodelling is required for the generation and maintenance of cancer-associated fibroblasts. Nat Cell Biol.

[9] Du X, Yu A, Tao W (2015). The non-canonical Hippo/Mst pathway in lymphocyte development and functions. Acta Biochim Biophys Sin Shanghai.

[10] Bardia A, Baselga J (2013). Neoadjuvant therapy as a platform for drug development and approval in breast cancer. Clin Cancer Res.

[11] Vici P, Mottolese M, Pizzuti L, Barba M, Sperati F, Terrenato I (2014). The Hippo transducer TAZ as a biomarker of pathological complete response in HER2-positive breast cancer patients treated with trastuzumab-based neoadjuvant therapy. Oncotarget.

[12] Foulkes WD, Smith IE, Reis-Filho JS (2010). Triple-negative breast cancer. N Engl J Med.

[13] Salgado R, Denkert C, Demaria S, Sirtaine N, Klauschen F, Pruneri G (2015). The evaluation of tumor-infiltrating lymphocytes (TILs) in breast cancer: recommendations by an International TILs Working Group 2014. Ann Oncol.

[14] Vici P, Di Benedetto A, Ercolani C, Pizzuti L, Di Lauro L, Sergi D (2015). Predictive significance of DNA damage and repair biomarkers in triple-negative breast cancer patients treated with neoadjuvant chemotherapy: an exploratory analysis. Oncotarget.

[15] Kim SK, Jung WH, Koo JS (2014). Yes-associated protein (YAP) is differentially expressed in tumor and stroma according to the molecular subtype of breast cancer. Int J Clin Exp Pathol.

[16] Min Kim H, Kim SK, Jung WH, Koo JS (2015). Metaplastic carcinoma show different expression pattern of YAP compared to triple-negative breast cancer. Tumour Biol.

[17] Lehn S, Tobin NP, Sims AH, Stål O, Jirström K, Axelson H, Landberg G (2014). Decreased expression of Yes-associated protein is associated with outcome in the luminal A breast cancer subgroup and with an impaired tamoxifen response. BMC Cancer.

[18] Chang HY, Nuyten DS, Sneddon JB, Hastie T, Tibshirani R, Sørlie T (2005). Robustness, scalability, and integration of a wound-response gene expression signature in predicting breast cancer survival. Proc Natl Acad Sci U S A.

[19] Denkert C, von Minckwitz G, Brase JC, Sinn BV, Gade S, Kronenwett R (2015). Tumor-infiltrating lymphocytes and response to neoadjuvant chemotherapy with or without carboplatin in human epidermal growth factor receptor 2-positive and triple-negative primary breast cancers. J Clin Oncol.

[20] Mao Y, Keller ET, Garfield DH, Shen K, Wang J (2013). Stromal cells in tumor microenvironment and breast cancer. Cancer Metastasis Rev.

[21] Dupont S, Morsut L, Aragona M, Enzo E, Giulitti S, Cordenonsi M (2011). Role of YAP/TAZ in mechanotransduction. Nature.

[22] Sorrentino G, Ruggeri N, Specchia V, Cordenonsi M, Mano M, Dupont S (2014). Metabolic control of YAP and TAZ by the mevalonate pathway. Nat Cell Biol.

[23] Azzolin L, Panciera T, Soligo S, Enzo E, Bicciato S, Dupont S (2014). YAP/TAZ incorporation in the β-catenin destruction complex orchestrates the Wnt response. Cell.

[24] Chan SW, Lim CJ, Guo K, Ng CP, Lee I, Hunziker W (2008). A role for TAZ in migration, invasion, and tumorigenesis of breast cancer cells. Cancer Res.

[25] Lai D, Ho KC, Hao Y, Yang X (2011). Taxol resistance in breast cancer cells is mediated by the hippo pathway component TAZ and its downstream transcriptional targets Cyr61 and CTGF. Cancer Res.

[26] Lei QY, Zhang H, Zhao B, Zha ZY, Bai F, Pei XH (2008). TAZ promotes cell proliferation and epithelial-mesenchymal transition and is inhibited by the hippo pathway. Mol Cell Biol.

[27] Skibinski A, Breindel JL, Prat A, Galván P, Smith E, Rolfs A (2014). The Hippo transducer TAZ interacts with the SWI/SNF complex to regulate breast epithelial lineage commitment. Cell Rep.

[28] Bendinelli P, Maroni P, Matteucci E, Luzzati A, Perrucchini G, Desiderio MA (2013). Hypoxia inducible factor-1 is activated by transcriptional co-activator with PDZ-binding motif (TAZ) versus WWdomain-containing oxidoreductase (WWOX) in hypoxic microenvironment of bone metastasis from breast cancer. Eur J Cancer.

[29] Overholtzer M, Zhang J, Smolen GA, Muir B, Li W, Sgroi DC (2006). Transforming properties of YAP, a candidate oncogene on the chromosome 11q22 amplicon. Proc Natl Acad Sci U S A.

[30] Wang X, Su L, Ou Q (2012). Yes-associated protein promotes tumour development in luminal epithelial derived breast cancer. Eur J Cancer.

[31] Chen Q, Zhang N, Gray RS, Li H, Ewald AJ, Zahnow CA, Pan D (2014). A temporal requirement for Hippo signaling in mammary gland differentiation, growth, and tumorigenesis. Genes Dev.

[32] Lamar JM, Stern P, Liu H, Schindler JW, Jiang ZG, Hynes RO (2012). The Hippo pathway target, YAP, promotes metastasis through its TEAD-interaction domain. Proc Natl Acad Sci U S A.

[33] Chen D, Sun Y, Wei Y, Zhang P, Rezaeian AH, Teruya-Feldstein J (2012). LIFR is a breast cancer metastasis suppressor upstream of the Hippo-YAP pathway and a prognostic marker. Nat Med.

[34] Yu SJ, Hu JY, Kuang XY, Luo JM, Hou YF, Di GH (2013). MicroRNA-200a promotes anoikis resistance and metastasis by targeting YAP1 in human breast cancer. Clin Cancer Res.

[35] Yuan M, Tomlinson V, Lara R, Holliday D, Chelala C, Harada T (2008). Yes-associated protein (YAP) functions as a tumor suppressor in breast. Cell Death Differ.

[36] Zhang H, Liu CY, Zha ZY, Zhao B, Yao J, Zhao S (2009). TEAD transcription factors mediate the function of TAZ in cell growth and epithelial-mesenchymal transition. J Biol Chem.

[37] Cui CB, Cooper LF, Yang X, Karsenty G, Aukhil I (2003). Transcriptional coactivation of bone-specific transcription factor Cbfa1 by TAZ. Mol Cell Biol.

[38] Hau JC, Erdmann D, Mesrouze Y, Furet P, Fontana P, Zimmermann C (2013). The TEAD4-YAP/TAZ protein-protein interaction: expected similarities and unexpected differences. Chembiochem.

