# Supplementary material for: Topographic expression of the Hippo transducers TAZ and YAP in triple-negative breast cancer treated with neoadjuvant chemotherapy
Source: J Exp Clin Cancer Res. 2016 Apr 2;35:62. doi: 10.1186/s13046-016-0338-7 (PMC4818869; doi:10.1186/s13046-016-0338-7)
Supplement: Additional file 1: Table S1. — Expression of TAZ and YAP in cancer cells, non-lymphocytic stromal cells, endothelial cells, and tumor-infiltrating lymphocytes. Subcellular localization of TAZ/YAP in tumors is also reported (N = 61). (DOCX 13 kb) [file 13046_2016_338_MOESM1_ESM.docx]

Supplementary Table 1: Expression of TAZ and YAP in cancer cells, non-lymphocytic stromal cells, endothelial cells, and tumor-infiltrating lymphocytes. Subcellular localization of TAZ/YAP in tumors is also reported (N=61).

|  | N(%) |
| --- | --- |
| **TAZ (tumor)** |  |
| Neg | 29 (47.5) |
| Pos | 32 (52.5) |
| 0 | 8 (13.1) |
| C | 21 (34.4) |
| N | 7 (11.5) |
| N/C | 25 (41.0) |
| **TAZ^stroma^** |  |
| Neg | 25 (41.0) |
| Pos | 36 (59.0) |
| **TAZ^end^** |  |
| Neg | 5 (8.2) |
| Pos | 56 (91.8) |
| **TAZ^TILs^** |  |
| Neg | 61 (100.0) |
| Pos | 0 (0.0) |
| **YAP (tumor)** |  |
| Neg | 33 (54.1) |
| Pos | 28 (45.9) |
| 0 | 3 (4.9) |
| C | 30 (49.2) |
| N | 3 (4.9) |
| N/C | 25 (41.0) |
| **YAP^stroma^** |  |
| Neg | 21 (34.4) |
| Pos | 40 (65.6) |
| **YAP^end^** |  |
| Neg | 18 (29.5) |
| Pos | 43 (70.5) |
| **YAP^TILs^** |  |
| Neg | 55 (90.2) |
| Pos | 6 (9.8) |
