# Supplementary material for: Topographic expression of the Hippo transducers TAZ and YAP in triple-negative breast cancer treated with neoadjuvant chemotherapy
Source: J Exp Clin Cancer Res. 2016 Apr 2;35:62. doi: 10.1186/s13046-016-0338-7 (PMC4818869; doi:10.1186/s13046-016-0338-7)
Supplement: Additional file 2: Table S2. — associations between TAZ/YAP, assessed in the tumor and in the microenvironment, and clinical-molecular features and pCR (N = 61). (DOCX 12 kb) [file 13046_2016_338_MOESM2_ESM.docx]

Supplementary table 2: associations between TAZ/YAP, assessed in the tumor and in the microenvironment, and clinical-molecular features and pCR (N=61)

|  | Stage | Ki-67 | Grade | pCR | TILs |
| --- | --- | --- | --- | --- | --- |
| TAZ^pos^ | **0.030** | 0.359 | 0.436 | 0.754 | 0.104 |
| TAZ^stroma^ | 0.829 | 0.952 | 0.582 | 0.830 | 0.690 |
| TAZ^end^ | 0.999* | 0.999* | 0.645* | 0.999* | 0.072* |
| TAZ^TILs^ | Not applicable | | | | |
| YAP^pos^ | 0.845 | 0.088 | **0.028** | 0.202 | 0.784 |
| YAP^stroma^ | 0.662 | 0.284 | 0.811 | 0.635 | 0.063 |
| YAP^end^ | 0.907 | 0.804 | 0.143 | 0.299 | 0.722 |
| YAP^TILs^ | 0.999* | 0.411* | 0.404* | 0.999* | 0.683* |

*Fisher's Exact Test
