# Supplementary material for: Topographic expression of the Hippo transducers TAZ and YAP in triple-negative breast cancer treated with neoadjuvant chemotherapy
Source: J Exp Clin Cancer Res. 2016 Apr 2;35:62. doi: 10.1186/s13046-016-0338-7 (PMC4818869; doi:10.1186/s13046-016-0338-7)
Supplement: Additional file 3: Table S3. — Pattern of recurrence in the 19 TNBC patients. (DOCX 12 kb) [file 13046_2016_338_MOESM3_ESM.docx]

Supplementary Table 3: Pattern of recurrence in the 19 TNBC patients

| **# Patient** | **Metastatic sites (N)** | **Site(s) of recurrence** |
| --- | --- | --- |
| 1 | 1 | Skin |
| 3 | 1 | Local recurrence |
| 4 | 1 | Bone |
| 6 | 1 | Lymph nodes |
| 12 | 1 | Local recurrence |
| 15 | 2 | Lung, liver |
| 17 | 2 | Lung, brain |
| 19 | 2 | Lung, bone |
| 22 | 2 | Lymph nodes, liver |
| 25 | 1 | Bone |
| 26 | 1 | Lymph nodes |
| 27 | 1 | Lymph nodes |
| 28 | 1 | Brain |
| 35 | 1 | Liver |
| 36 | 1 | Local recurrence |
| 46 | 3 | Lymph nodes, liver, bone |
| 47 | 1 | Liver |
| 50 | 1 | Lung |
| 55 | 2 | Lung, liver |
